# Delayed Genetic Diagnosis of a Rare Presentation of Overlapping Peripheral Myelin Protein 22 (PMP22) Neuropathies

**DOI:** 10.7759/cureus.112744

**Published:** 2026-07-15

**Authors:** Hemant K Pandey, Kinsey J Gudenkauf, Visweshwar Swaminathan

**Affiliations:** 1 Neurology, Brain and Spine Center, Chandler, USA; 2 Neurology, AT Still University KCOM (Kirksville College of Osteopathic Medicine), Mesa, USA; 3 Neurology, Arizona State University, Tempe, USA

**Keywords:** charcot-marie-tooth disease, genetic testing delay, hereditary neuropathy, hereditary pressure palsies, inherited demyelinating neuropathy, pmp22 mutation

## Abstract

Peripheral Myelin Protein 22 (PMP22) is a small integral membrane glycoprotein that is essential for the formation and maintenance of myelin architecture in the peripheral nervous system. Variations of the PMP22 gene can result in a variety of genetic conditions, with some of the most notable being Charcot-Marie-Tooth disease (CMT) as well as Hereditary Neuropathy with Liability to Pressure Palsies (HNPP). The patient had a strong family history of CMT, and she began experiencing recurrent foot drop, sensory loss, extremity weakness, and frequent falls; however, initial genetic testing did not reveal any mutation in the PMP22 gene. This case features the diagnostic challenges associated with inherited neuropathies when clinical suspicion is high but genetic results are initially inconclusive. It also emphasizes the importance of repeat or expanded genetic testing to ensure a timely diagnosis with appropriate management and family genetic counseling.

## Introduction

Charcot-Marie-Tooth disease (CMT) is an inherited neuropathy characterized by progressive distal muscle weakness, neuropathy, and foot deformities such as pes cavus and hammer toe deformities [1]. A mutation in the Peripheral Myelin Protein 22 (PMP22) gene, most commonly a duplication, is a common genetic cause of this disorder, in which the proteins of the myelin sheath are affected [1]. Hereditary Neuropathy with Liability to Pressure Palsies (HNPP) is another manifestation of this pathogenic variant, typically caused by a deletion on the PMP22 gene, that produces recurrent focal neuropathies triggered by compression or minor trauma [2]. Certain mutations in the PMP22 gene can embody overlapping features of both CMT and HNPP, depending on the mutation’s location and molecular effect. Diagnosis of these conditions can be challenging due to inaccurate genetic testing, limited access to testing, or the presence of confounding conditions.

This case describes a patient who presented with classic clinical features of CMT, including longstanding distal weakness, recurrent foot drop, sensory deficits, and characteristic foot deformities, in the setting of a strong family history of inherited neuropathy. Despite a highly suggestive clinical presentation, initial genetic testing was found to be negative, prompting further extensive evaluation for other causes.

## Case presentation

A 68-year-old woman with longstanding neuropathic symptoms and a family history of CMT presented with multiple falls over the preceding year. These included one episode associated with complete loss of consciousness and another involving partial loss of consciousness accompanied by transient vision and speech changes. She also reported recurrent right-sided foot drop and progressive weakness involving both the right upper and lower extremities. Her medical history was notable for bilateral carpal tunnel release, chronic migraines, tricuspid regurgitation, and significant weight loss following bariatric surgery without improvement in neuropathic symptoms. She additionally described symptoms consistent with CMT beginning during adolescence. Neurological examination revealed pes cavus and hammer toe deformities. Sensory testing demonstrated moderately severe vibration loss in the lower extremities, predominantly on the right side, along with sensory changes involving the right wrist and an area inferior to the right knee. Strength testing revealed significant right-sided weakness, most pronounced in right hand grip strength, right iliopsoas during hip flexion, and right tibialis anterior during ankle dorsiflexion. Gait evaluation demonstrated subtle drift and mild clumsiness attributed to foot drop.

**Figure 1:**
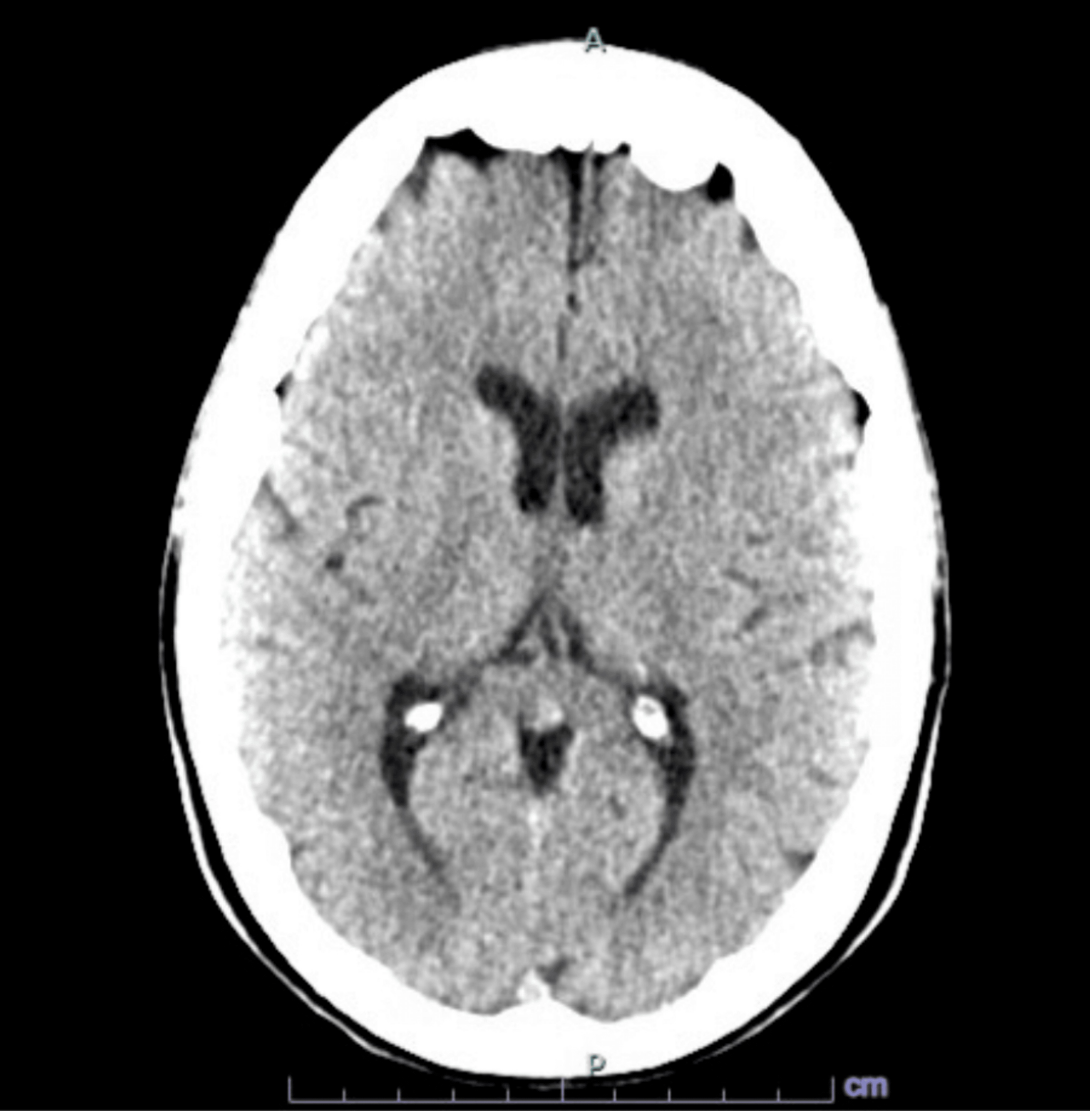
Axial non-contrast CT of head No acute intracranial hemorrhage, mass effect, or midline shift noted. Gray-white matter differentiation is preserved.

Cardiac evaluation and non-contrast computed tomography (CT) of the head were unremarkable (Figure 1).

**Figure 2:**
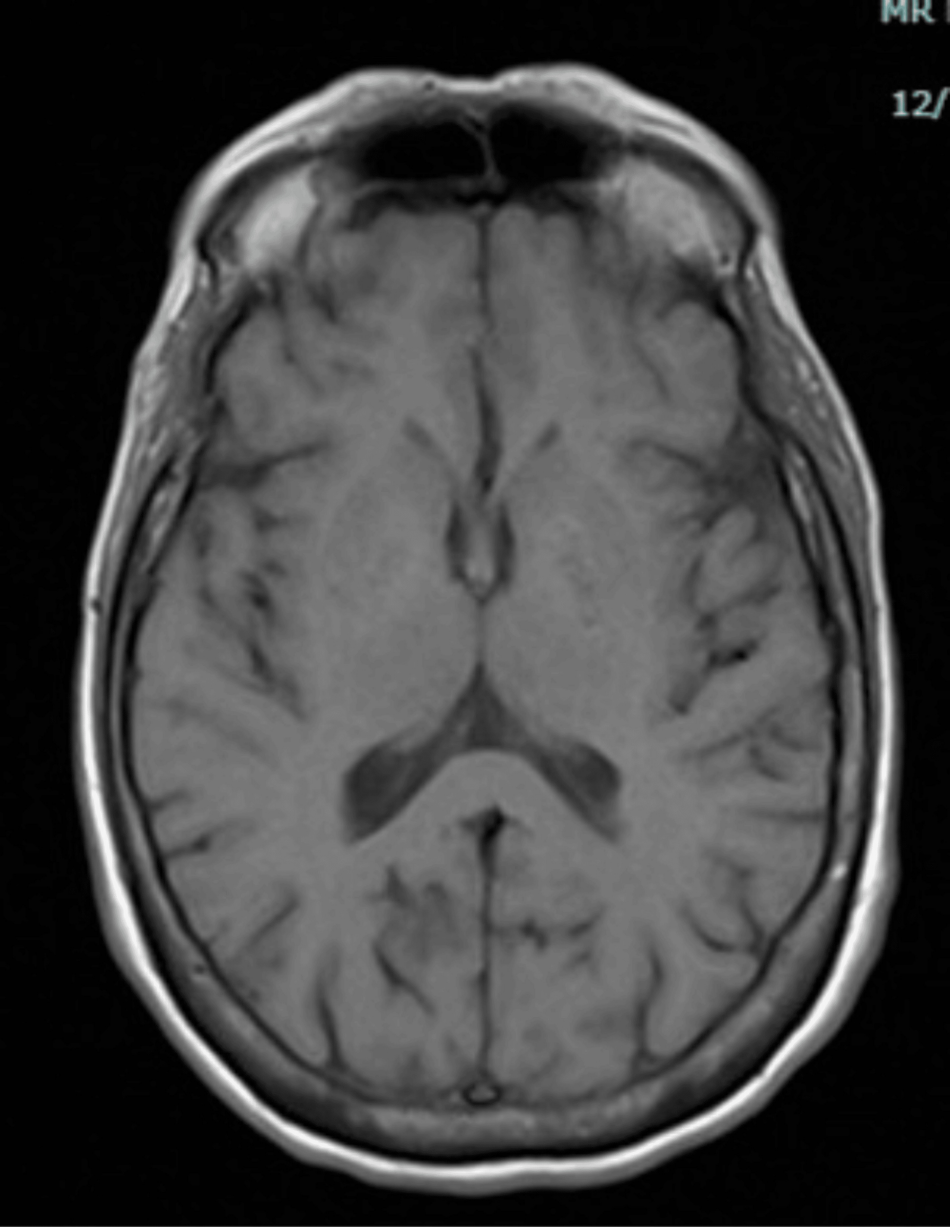
Axial T1-weighted MRI of the brain Mild chronic nonspecific white matter changes, most commonly reflecting chronic microvascular ischemic change. No acute intracranial abnormality identified.

Magnetic resonance imaging (MRI) of the brain demonstrated only mild chronic white matter changes without evidence of acute intracranial pathology (Figure 2).

**Figure 3:**
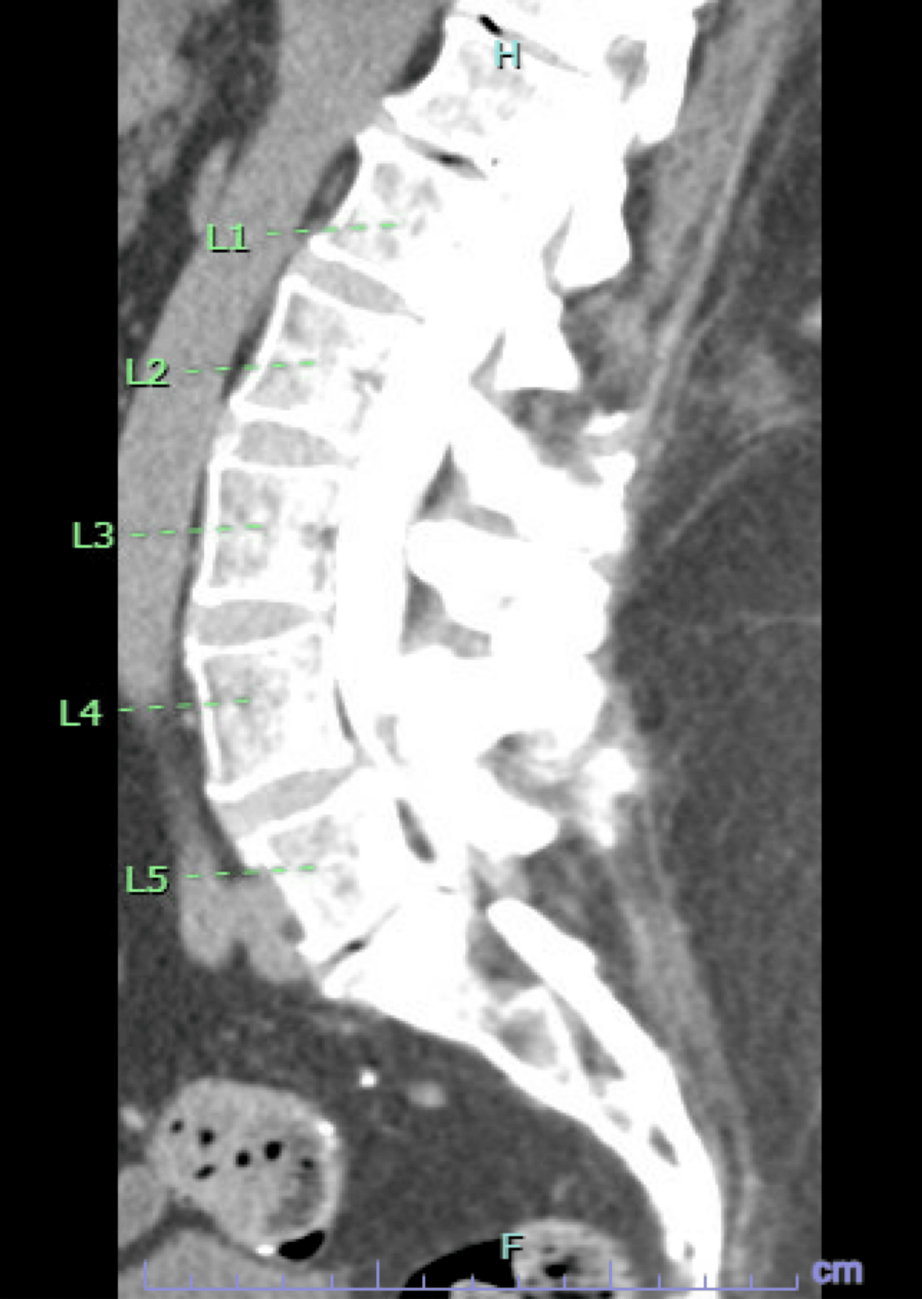
Sagittal lumbar spine CT with contrast Multifactorial moderate to severe central canal stenosis at L4-L5. Negative for neural foraminal stenosis. Degenerative disc disease with vacuum phenomenon T12-L1 and L5-S1. Negative for focal disc herniation, central canal or neural foraminal stenosis.

CT of the lumbar spine with contrast demonstrated moderate central canal stenosis at L4-L5 and degenerative disc disease with vacuum phenomenon at T12-L1 and L5-S1 (Figure 3).

Although these degenerative spinal findings could contribute to localized symptoms, they were insufficient to explain the patient's diffuse, symmetric sensorimotor deficits. Electrodiagnostic testing of the upper extremities demonstrated prolonged distal peak latencies and decreased conduction velocities of the bilateral median sensory nerves, with absent bilateral ulnar sensory responses (Table 1).

**Table 1:** Upper extremities nerve conduction studies (anti-sensory summary)

Stimulation site	Not recorded	Peak (ms)	Normal peak (ms)	Peak-to-peak amplitude (μV)	Normal peak-to-peak amplitude (μV)	Site 1	Site 2	Delta-0 (ms)	Distance (cm)	Velocity (m/s)	Normal velocity (m/s)
Left median anti-sensory (2nd digit) 30.5°C											
Attempt 1	N/A	5.8	<3.6	11.6	>10	Wrist	2nd digit	4.5	13.0	28.9	>49
Attempt 2	N/A	6.0	<3.6	12.8	>10	Wrist	2nd digit	4.6	13.0	28.3	>49
Attempt 3	N/A	6.0	<3.6	14.0	>10	Wrist	2nd digit	4.7	13.0	27.7	>49
Right median anti-sensory (2nd digit) 30.8°C											
Attempt 1	N/A	5.4	<3.6	11.8	>10	Wrist	2nd digit	4.3	13.0	30.2	>49
Attempt 2	N/A	5.6	<3.6	14.4	>10	Wrist	2nd digit	4.3	13.0	30.2	>49
Attempt 3	N/A	5.7	<3.6	12.3	>10	Wrist	2nd digit	4.3	13.0	30.2	>49
Left ulnar anti-sensory (5th digit) 30.6°C											
Attempt 1	Not recorded	N/A	<3.2	N/A	>15	Wrist	5th digit	N/A	11.0	N/A	>49
Attempt 2	Not recorded	N/A	<3.2	N/A	>15	Wrist	5th digit	N/A	11.0	N/A	>49
Attempt 3	Not recorded	N/A	<3.2	N/A	>15	Wrist	5th digit	N/A	11.0	N/A	>49
Right ulnar anti-sensory (5th digit) 30.7°C											
Attempt 1	Not recorded	N/A	<3.2	N/A	>15	Wrist	5th digit	N/A	11.0	N/A	>49
Attempt 2	Not recorded	N/A	<3.2	N/A	>15	Wrist	5th digit	N/A	11.0	N/A	>49
Attempt 3	Not recorded	N/A	<3.2	N/A	>15	Wrist	5th digit	N/A	11.0	N/A	>49

Bilateral median motor nerves and the right ulnar motor nerve also demonstrated prolonged distal onset latencies (Table 2).

**Table 2:** Upper extremities nerve conduction studies (motor summary)

Stimulation site	Not recorded	Onset (ms)	Normal onset (ms)	Amplitude origin to proximal site (mV)	Normal amplitude origin to proximal site (mV)	Site 1	Site 2	Delta-0 (ms)	Distance (cm)	Velocity (m/s)	Normal velocity (m/s)
Left median motor (abductor pollicis brevis) 29.9°C											
Wrist	N/A	5.6	<4.3	5.6	>5.0	Elbow	Wrist	4.7	21.0	44.7	>49
Elbow	N/A	10.3	N/A	4.8	N/A	N/A	N/A	N/A	N/A	N/A	N/A
Right median motor (abductor pollicis brevis) 30.6°C											
Wrist	N/A	5.4	<4.3	6.7	>5.0	Elbow	Wrist	3.9	20.0	51.3	>49
Elbow	N/A	9.3	N/A	6.7	N/A	N/A	N/A	N/A	N/A	N/A	N/A
Left ulnar motor (abductor digiti minimi) 30.4°C											
Wrist	N/A	3.8	<4.2	6.0	>5.9	B Elbow	Wrist	4.3	22.0	51.2	>49
B Elbow	N/A	8.1	N/A	5.4	N/A	A Elbow	B Elbow	1.9	11.0	57.9	>49
A Elbow	N/A	10.0	N/A	5.3	N/A	N/A	N/A	N/A	N/A	N/A	N/A
Right ulnar motor (abductor digiti minimi) 30.8°C											
Wrist	N/A	5.2	<4.2	6.9	>5.9	B Elbow	Wrist	4.4	22.0	50.0	>49
B Elbow	N/A	9.6	N/A	6.7	N/A	A Elbow	B Elbow	2.0	11.0	55.0	>49
A Elbow	N/A	11.6	N/A	6.6	N/A	N/A	N/A	N/A	N/A	N/A	N/A

Needle electromyography of the right abductor pollicis brevis showed slightly increased polyphasic motor unit potentials (Table 3), consistent with chronic denervation and reinnervation.

**Table 3:** Electromyography of upper extremities

Side	Muscle	Nerve	Root	Insertional activity	Fibrillation potentials	Positive sharp waves	Complex repetitive discharges	Myotonic	Myokymia	Fasiculations	Amplitude	Duration	Polyphasic	Recruitment	Interference pattern
Right	Deltoid	Axillary	C5-6	Normal	Normal	Normal	Normal	Normal	Normal	Normal	Normal	Normal	0	Normal	Normal
Right	Biceps	Musculocutaneous	C5-6	Normal	Normal	Normal	Normal	Normal	Normal	Normal	Normal	Normal	0	Normal	Normal
Right	Triceps	Radial	C6-7-8	Normal	Normal	Normal	Normal	Normal	Normal	Normal	Normal	Normal	0	Normal	Normal
Right	1st dorsal interosseous	Ulnar	C8-T1	Normal	Normal	Normal	Normal	Normal	Normal	Normal	Normal	Normal	0	Normal	Normal
Right	Abductor pollicis brevis	Median	C8-T1	Normal	Normal	Normal	Normal	Normal	Normal	Normal	Normal	Normal	1+	Normal	Normal

Electrodiagnostic testing of the lower extremities revealed absent sensory responses bilaterally. Bilateral peroneal motor nerves demonstrated prolonged distal onset latencies, reduced compound muscle action potential amplitudes, and slowed conduction velocities, while bilateral tibial motor nerves also exhibited decreased conduction velocities (Table 4).

**Table 4:** Lower extremities nerve conduction studies (motor summary)

Stimulation site	Not recorded	Onset (ms)	Normal onset (ms)	Amplitude origin to proximal site (mV)	Normal amplitude origin to proximal site (mV)	Site 1	Site 2	Delta-0 (ms)	Distance (cm)	Velocity (m/s)	Normal velocity (m/s)
Left peroneal motor (extensor digitorum brevis) 28.2°C											
Ankle	N/A	6.3	<6.1	1.3	>2.5	Below fibular head	Ankle	11.7	31	26	>38
Below fibular head	N/A	18.0	N/A	0.9	N/A	Popliteal	Below fibular head	2.3	5	22	>40
Popliteal	N/A	20.3	N/A	0.7	N/A	N/A	N/A	N/A	N/A	N/A	N/A
Right peroneal motor (extensor digitorum brevis) 28.1°C											
Ankle	N/A	10.5	<6.1	0.4	>2.5	Below fibular head	Ankle	14.0	31	22	>38
Below fibular head	N/A	24.5	N/A	0.3	N/A	Popliteal	Below fibular head	1.5	5	33	>40
Popliteal	N/A	26.0	N/A	0.3	N/A	N/A	N/A	N/A	N/A	N/A	N/A
Left tibial motor (abductor hallucis brevis) 28.2°C											
Ankle	N/A	4.4	<6.1	4.9	>3.0	Knee	Ankle	10.8	34	31	>35
Knee	N/A	15.2	N/A	3.1	N/A	N/A	N/A	N/A	N/A	N/A	N/A
Right tibial motor (abductor hallucis brevis) 28.1°C											
Ankle	N/A	5.1	<6.1	3.5	>3.0	Knee	Ankle	10.8	34	31	>35
Knee	N/A	15.9	N/A	0.7	N/A	N/A	N/A	N/A	N/A	N/A	N/A

Needle examination demonstrated increased insertional and spontaneous activity in the right anterior tibialis and right gastrocnemius muscles, with moderately increased polyphasic motor unit potentials in the right anterior tibialis and left anterior tibialis (Table 5), indicating ongoing denervation with chronic reinnervation.

**Table 5:** Electromyography of lower extremities

Side	Muscle	Nerve	Root	Insertional activity	Fibrillation potentials	Positive sharp waves	Complex repetitive discharges	Myotonic	Myokymia	Fasiculations	Amplitude	Duration	Polyphasic	Recruitment	Interference pattern
Right	Vastus lateralis	Femoral	L2-4	Normal	Normal	Normal	Normal	Normal	Normal	Normal	Normal	Normal	0	Normal	Normal
Right	Anterior tibialis	Peroneal: deep branch	L4-5	Increased	3+	Normal	Normal	Normal	Normal	Normal	Normal	Normal	2+	Normal	Normal
Right	Gastrocnemius	Tibial	S1-2	Increased	1+	Normal	Normal	Normal	Normal	Normal	Normal	Normal	0	Normal	Normal
Right	Iliopsoas	Femoral	L2-3	Normal	Normal	Normal	Normal	Normal	Normal	Normal	Normal	Normal	0	Normal	Normal
Right	Biceps femoris	Sciatic	L5-S1	Normal	Normal	Normal	Normal	Normal	Normal	Normal	Normal	Normal	0	Normal	Normal
Left	Anterior tibialis	Peroneal: deep branch	L4-5	Normal	Normal	Normal	Normal	Normal	Normal	Normal	Normal	Normal	2+	Normal	Normal

Overall, these electrodiagnostic findings are most consistent with a chronic, length-dependent hereditary sensorimotor polyneuropathy characterized by both demyelinating features, such as the marked slowing of conduction velocities and prolonged distal latencies, and secondary axonal loss, demonstrated by reduced motor amplitudes, absent sensory responses, and chronic neurogenic changes on needle electromyography. The greater severity in the distal lower extremities is characteristic of CMT, reflecting the typical length-dependent progression of the disorder. The absence of acute denervation in proximal muscles and lack of electrodiagnostic evidence for a focal radiculopathy further support a diffuse hereditary neuropathy.

Extensive laboratory evaluation for secondary causes of neuropathy was unrevealing. Normal or negative results were obtained for methylmalonic acid, erythrocyte sedimentation rate, complete blood count, fasting and postprandial glucose, vitamin B12, thyroid-stimulating hormone with reflex free T4, folate, comprehensive metabolic panel, syphilis serology, Sjögren’s antibodies, vitamin B1, and celiac disease panel. Serum protein electrophoresis was negative except for a slightly decreased IgM level of 44 mg/dL. Initial genetic testing using Multiplex Ligation-dependent Probe Amplification for PMP22 deletion/duplication was negative.

Initial concern for stroke was prompted by the patient's acute onset of right-sided weakness and gait impairment, but was excluded following an unremarkable cardiac evaluation, negative non-contrast CT of the head, and brain MRI demonstrating only mild chronic white matter changes without evidence of acute infarction. Furthermore, her longstanding history of recurrent peripheral weakness, chronic foot drop, pes cavus, hammer toes, progressive sensory loss, and electrodiagnostic evidence of a length-dependent polyneuropathy was not consistent with an acute cerebrovascular event or other central nervous system process. Lumbar radiculopathy was considered based on CT findings demonstrating moderate central canal stenosis at L4-L5 with degenerative disc disease at T12-L1 and L5-S1, as well as electromyographic evidence of right-sided denervation involving the tibialis anterior and gastrocnemius muscles. Although these findings could explain focal lower extremity symptoms, lumbar pathology alone could not account for the chronic bilateral distal weakness, diffuse sensory abnormalities, upper extremity involvement, pes cavus, hammer toes, or widespread abnormalities on nerve conduction studies, making an isolated compressive radiculopathy unlikely to explain the overall clinical picture. An acquired polyneuropathy was also considered because electrodiagnostic testing demonstrated a mixed large-fiber axonal and demyelinating sensorimotor neuropathy. Potential metabolic, nutritional, autoimmune, toxic, infectious, and paraproteinemic etiologies were systematically evaluated through laboratory testing, including appropriate metabolic and serologic studies, all of which were unrevealing. Additionally, the gradual onset beginning in adolescence and strong multigenerational family history argued against an acquired neuropathy. Entrapment neuropathy was considered given her prior bilateral carpal tunnel release and electrodiagnostic evidence of bilateral median neuropathy at the wrists with absent ulnar sensory responses. However, entrapment neuropathies are common in hereditary neuropathies and would not explain the diffuse, length-dependent sensorimotor deficits involving both the upper and lower extremities, characteristic foot deformities, or extensive family history of CMT. Collectively, the patient's clinical phenotype, electrodiagnostic findings, and family history were most consistent with an underlying hereditary neuropathy, which was ultimately confirmed by repeat genetic testing identifying a pathogenic PMP22 variant.

Because of the strong clinical suspicion for an inherited neuropathy based on adolescent symptom onset, physical examination findings, electrodiagnostic evidence of mixed demyelinating and axonal neuropathy, and a family history involving nine cousins affected by Charcot-Marie-Tooth disease, repeat genetic testing using next-generation sequencing was performed one year after the initial negative genetic evaluation. This identified a heterozygous autosomal dominant PMP22 nonsense variant, c.396C>A (p.Tyr132*), which was subsequently confirmed on repeat testing. According to the Athena Insights pathogenicity assessment, this variant was classified as pathogenic based on the following findings. Although the variant is not predicted to cause loss of protein expression through nonsense-mediated decay, pathogenic variants affecting the same region of PMP22 have previously been associated with HNPP, supporting the functional importance of this region. Furthermore, PMP22 is a well-established disease gene for autosomal dominant CMT and HNPP. The patient's phenotype was highly consistent with HNPP, demonstrating genotype-phenotype concordance, and the variant was absent from large population databases, including the Genome Aggregation Database, arguing against a benign polymorphism.

Based on her clinical presentation, family history, physical examination findings, and follow-up confirmatory genetic testing, she was diagnosed with CMT with overlapping HNPP related to the PMP22 pathogenic gene variant. Management focused on supportive and symptomatic treatment. She was prescribed an ankle-foot orthosis to address right foot drop and gabapentin 300 mg daily for neuropathic pain. Given the hereditary nature of PMP22 gene mutations and the presence of neuropathic symptoms in her daughter, genetic counseling and screening were recommended. She was also referred to a neuromuscular specialist for ongoing management.

At follow-up, the patient continued to experience right foot drop but reported improved ambulation with consistent ankle-foot orthosis use. Sensory deficits and vibration loss persisted in previously documented distributions, and mild residual weakness remained in the right gastrocnemius and tibialis anterior muscles. The patient demonstrated understanding of her diagnosis and the importance of family screening. This case also notes the broader clinical implication that persistent phenotype-driven suspicion should prompt re-evaluation with evolving genetic technologies when initial molecular testing is inconclusive, particularly in patients with a compelling clinical presentation and family history.

## Discussion

CMT and HNPP are allelic disorders caused by pathogenic variants in the PMP22 gene; however, only approximately 20-64% of patients with CMT have the classic PMP22 duplication [2]. CMT is characterized by progressive distal muscle weakness and atrophy, sensory loss, and foot deformities that typically begin during adolescence or early adulthood, consistent with this patient's teenage onset of symptoms. In contrast, HNPP is characterized by recurrent focal neuropathies precipitated by minor trauma or nerve compression, most commonly involving the peroneal or ulnar nerves in approximately 70% of cases [3]. The coexistence of longstanding CMT features with recurrent focal neuropathies suggested an overlapping hereditary neuropathy in this patient.

Despite this compelling phenotype, initial genetic testing was negative for a PMP22 abnormality. Given the patient's characteristic clinical features, including pes cavus, hammer toes, recurrent foot drop, supportive electrodiagnostic findings, and a strong family history of CMT, repeat genetic evaluation was pursued. Next-generation sequencing identified a heterozygous pathogenic PMP22 nonsense variant, c.396C>A (p.Tyr132*), which was subsequently confirmed on repeat analysis. False-negative genetic results may occur because of limitations of earlier testing panels, evolving molecular technologies, or variants not detectable by the initial assay. In one study evaluating hereditary neuropathies, repeat genetic assessment established a molecular diagnosis in 44% of clinically diagnosed patients whose initial testing had been unrevealing [4]. These findings emphasize the importance of reconsidering genetic testing when the clinical phenotype remains highly suggestive despite negative initial results.

The patient's presentation also posed a significant diagnostic challenge because her unilateral weakness initially raised concern for an acute cerebrovascular event. Cardiac comorbidities, lumbar degenerative disease, and significant weight loss following bariatric surgery further broadened the differential diagnosis. Electrodiagnostic studies were therefore instrumental in distinguishing an inherited neuropathy from alternative etiologies. The combination of demyelinating median neuropathy, absent ulnar sensory responses, diffuse lower extremity abnormalities with both axonal and demyelinating features, chronic foot drop, and multifocal neuropathies was far more consistent with a hereditary sensorimotor neuropathy than with isolated entrapment neuropathy or lumbar radiculopathy [5].

The discrepancy between the initial and repeat genetic evaluations likely reflects the differing capabilities of the testing methodologies employed. Multiplex ligation-dependent probe amplification (MLPA), commonly used as a first-line test for PMP22-related neuropathies, is highly sensitive for detecting the common 1.5-Mb PMP22 duplication responsible for CMT1A and the reciprocal deletion that accounts for most cases of HNPP. However, MLPA is not designed to detect single-nucleotide variants or small insertions and deletions within the PMP22 coding sequence. In contrast, next-generation sequencing (NGS) provides comprehensive sequence analysis and was able to identify the pathogenic nonsense variant c.396C>A (p.Tyr132*) in this patient. This case highlights the complementary roles of MLPA and NGS in the evaluation of hereditary neuropathies and reinforces the importance of pursuing sequence-based testing when copy-number analysis is negative despite a compelling phenotype. Recognition of these uncommon PMP22 sequence variants expands the known molecular spectrum of CMT and HNPP and may prevent delayed diagnosis, inappropriate counseling, and missed opportunities for family screening.

## Conclusions

This case underscores the diagnostic challenges of inherited neuropathies when clinical features strongly suggest a genetic etiology but initial testing is unrevealing. In a patient with longstanding symptoms, characteristic examination findings, supportive electrodiagnostic studies, and a compelling family history, an initial negative PMP22 result delayed definitive diagnosis and appropriate counseling. Repeat and expanded genetic testing ultimately confirmed a pathogenic PMP22 variant, allowing accurate classification of her overlapping CMT and HNPP. Clinicians should maintain a high index of suspicion in similar presentations and consider reassessment with updated genetic testing when results are discordant with the clinical picture, as timely diagnosis has important implications for management, prognosis, and family screening. Furthermore, this case emphasizes the importance of recognizing PMP22 sequence variants in addition to the more commonly detected copy-number abnormalities, expanding the understanding of the genetic spectrum underlying hereditary neuropathies and reinforcing the value of comprehensive molecular testing.
